# Prednisone and Vincristine for the Treatment of Pediatric Rosai–Dorfman Disease: A Case Report

**DOI:** 10.1155/carm/5059027

**Published:** 2025-12-18

**Authors:** Jianxin Dun, Qun Hu, Aiguo Liu, Yaqin Wang, Ai Zhang

**Affiliations:** ^1^ Institute of Pediatric Hematology, Tongji Hospital, Tongji Medical College, Huazhong University of Science and Technology, Wuhan, 430030, China, hust.edu.cn

**Keywords:** chemotherapy, pathology, Rosai–Dorfman disease

## Abstract

**Purpose:**

Pediatric Rosai–Dorfman disease (RDD) is extremely rare, and the current treatment plan is not unified. We hope that our study provides new ideas for the clinical treatment of RDD.

**Methods:**

We report a case in which RDD was successfully treated with prednisone and vincristine, resulting in regression of enlarged lymph nodes. In this study, informed consent forms were obtained from participants for their involvement in the research and publication of relevant case details. Moreover, this study aimed to conceal personal patient information.

**Results:**

The child underwent two pathological examinations before a definitive diagnosis was made. Percutaneous fine‐needle aspiration biopsy was performed on his first admission, and pathological examination showed a purulent granulomatous reaction under a microscope; however, antibiotic treatment was ineffective. The second pathological biopsy performed at the time of surgery showed RDD. After treatment with vincristine and prednisone, the neck mass significantly shrank and did not increase in size after the treatment was stopped. The patient remains under follow‐up.

**Conclusions:**

Sufficient pathological tissue samples were obtained via surgical biopsy to diagnose RDD. Treatment with chemotherapy, including prednisone and vincristine, can induce remission of RDD.

## 1. Introduction

Rosai–Dorfman disease (RDD) is a rare form of non‐Langerhans cell histiocytosis. The prevalence of RDD is approximately 1:20,0000, with a mean age of onset of 20.6 years [1]. Classic RDD is characterized by bilateral cervical lymphadenopathy, and 43% of patients have extranodal changes in the skin, upper respiratory tract, bones, orbit, brain, and spinal cord [2]. RDD can occur as an isolated disorder or in association with autoimmune or malignant neoplastic disease [1]. RDD is characterized by sinusoidal enlargement of large histiocytes with pale or “watery” cytoplasm, large, light‐stained nuclei, and prominent nuclei and is immunophenotyped positive for CD68+ and S100+ and negative for CD1a [3].

Herein, we report the case of a 9‐year‐old boy who presented with enlarged cervical lymph nodes. He went through two biopsies of the cervical lymph nodes before being diagnosed with RDD. After the diagnosis was confirmed, the patient was treated with 5 months of prednisone and vincristine for 6 times. The mass was significantly reduced, and he is still being followed up.

## 2. Case Presentation

A 9‐year‐old boy was admitted to our hospital in October 2023 because of enlarged lymph nodes in the neck. The symptoms had been present for 1 month and had worsened over the past week, with enlarged lymph nodes on the right side. The enlarged lymph nodes were hard in texture, tender, and not easy to move without increased local skin temperature. Routine blood tests revealed elevated white blood cell, neutrophil, and platelet counts. The white blood cell count was 19.76 × 10^9^/L, the neutrophil count was 16.79 × 10^9^/L, and the platelet count was 706 × 10^9^/L. In addition, the IL‐2R, LR‐6, and TNF‐α levels were elevated. Neck MRI performed at the previous hospital showed enlarged lymph nodes on both sides, particularly on the right side. The largest lymph node measured approximately 19 × 47 mm on the coronal image.

**Figure 1:**
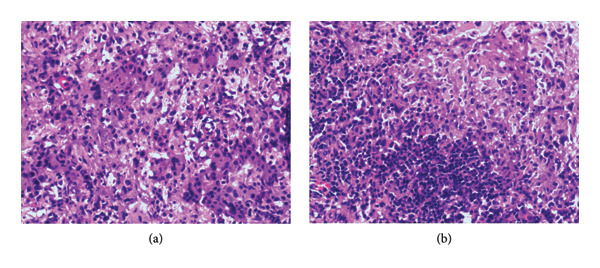
Lymph node puncture pathology by percutaneous fine‐needle aspiration biopsy (HE staining × 400).

Percutaneous fine‐needle aspiration biopsy was performed at his first admission, and pathological examination showed a purulent granulomatous reaction under the microscope (Figure 1), and immunohistochemistry (IHC) showed follicular CD20+, PAX5+, CD79α+, CD21 (FDC+), interfollicular zone CD3+, plasma cell MUMI (scattered +), EMA (plasma cell +), CD68 (histiocyte +), LCA+, CD30 (5% +, positive control +), TdT ‐, GATA‐3‐, ALK1A4‐, Ki‐67 (inflammatory cells high), PAS‐, acid‐fast staining‐, and EBER‐. We performed mNGS for pathogenic microorganisms, but the results were negative. Flow cytometry was used to detect the biopsy samples, and no abnormalities were found.

**Figure 2:**
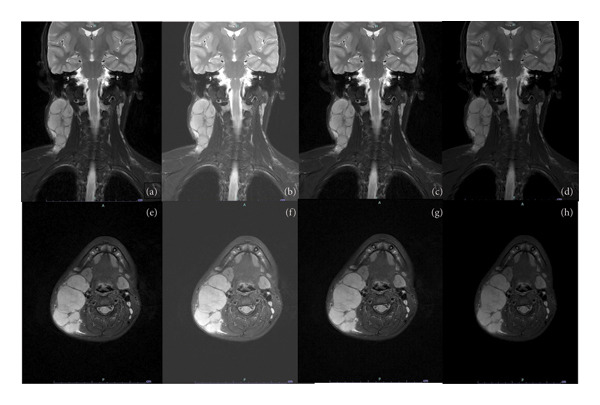
Radiographic features of RDD: (a) coronal original image, (b) coronal head FLAIR image, (c) coronal T1 image, (d) coronal T2 image, (e) axial original image, (f) axial head FLAIR image, (g) axial T1 image, and (h) axial T2 image.

Based on the pathological results, the patient was primarily diagnosed with septic lymphadenitis and treated with azithromycin 0.25 g once daily. However, a month later, he returned to our hospital with further enlargement of the neck lymph nodes. After the second admission, neck magnetic resonance diffusion‐weighted imaging (MRDWI) and fluorine‐18 fluorodeoxyglucose positron emission tomography/computed tomography (18F FDG PET‐CT) were performed. MRDWI of the masses showed an isointense signal on T1WI, a hyperintense signal on T2WI, and restricted diffusion on DWI, with a larger signal of approximately 37 × 34 mm on the right neck (Figure 2). PET‐CT showed several enlarged and fused lymph nodes in the right neck and right supraclavicular fossa with high glucose uptake, and no enlarged lymph nodes or abnormal uptake was found in the remaining scanning sites.

**Figure 3:**
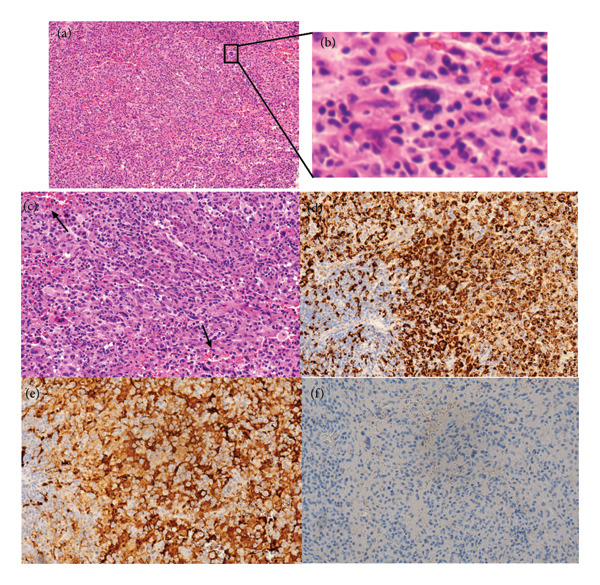
Surgical excision biopsy pathology: (a) histopathological microscopy (HE staining × 100), (b) emperipolesis (HE staining × 100), (c) dilated lymphatic sinuses (HE staining × 200), (d) CD68+ by IHC (× 200), (e) S‐100+ by IHC (× 200), and (f) Langerin‐ by IHC (× 200).

The patient underwent a second surgical excision biopsy of the right neck. Finally, we obtained the pathological diagnosis of RDD using IHC as follows: CD68+, CD163+, S‐100+, CyclinD1 (scattered +), CD1α‐, CD21‐, CD23‐, CD35‐, Langerin‐, Braf (V600E)‐, BRAF‐, SSTR‐, CD3‐, CD20‐, CD20‐, Ki‐67 (scattered +), and EBER CISH‐ (Figure 3).

**Figure 4:**
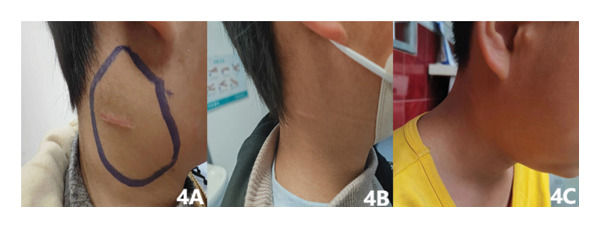
Lymph node changes in the right side of the child’s right neck: (A) pretreatment, (B) before vincristine, and (C) most recent follow‐up.

Oral prednisone was administered at an initial dose of 20 mg twice daily (1.5 mg/kg/d). In the first 2 weeks, the neck lesion gradually reduced. Oral prednisone was then decreased to 10 mg twice daily, and no obvious reduction in the lesion was observed. Cervical ultrasound before and after prednisone reduction also showed no significant changes in enlarged lymph nodes. In addition, the boy experienced adverse effects of glucocorticoids, including Cushing’s face and weight gain. Therefore, we decided to reduce prednisone to 5 mg twice daily combined with vincristine treatment. Vincristine was administered at a dose of 1.5 mg/m^2^ each time and once a week for a total of six times. Oral prednisone was reduced every 2‐3 weeks and stopped in June 2024 (Figure 4). After 2 months, cervical ultrasonography was performed regularly, and the results are listed in Table 1.

**Table 1:** Regular review of cervical ultrasound.

Dates	Cervical ultrasound		
	Max (mm)	Boundaries	Echogenicity
2023/11/17	71 × 40	Clear	Heterogeneous
2024/01/25	46 × 15	Clear	Heterogeneous
2024/02/26	32 × 17	Clear	Heterogeneous
2024/04/04	24 × 14	Clear	Hypoechogenicity
2024/06/17	27 × 11	Clear	Hypoechogenicity
2024/08/03	15 × 8	Clear	Hypoechogenicity

## 3. Discussion

RDD, also known as sinus histiocytosis with massive lymphadenopathy, is a rare benign histiocytic proliferative disorder. It was first described by Juan Rosai and Ronald Dorfman in 1969, hence the name [4]. However, the exact cause of RDD remains unclear. Some studies have detected the DNA of the Epstein–Barr virus, simplex virus, and other viral tissue samples, but these findings are not consistent [5].

Most classic (nodal) RDDs present as significantly enlarged bilateral painless cervical lymph nodes, with or without fever, night sweats, and weight loss. Some may also involve mediastinal, axillary, and inguinal lymph nodes [6]. In this case, the child had enlarged cervical lymph nodes bilaterally, tenderness on the right side, and no fever during the course of the disease. A definitive RDD diagnosis requires pathological biopsy. The percutaneous fine‐needle aspiration biopsy, however, carries a risk of missed diagnosis primarily due to the lack of a complete tissue architecture for evaluation, compounded by inadequate sampling [7]. In this case, the patient initially underwent a lymph node aspiration biopsy, which led to a primary diagnosis of lymphadenitis. The diagnosis was not confirmed until the second biopsy was performed. The characteristic histopathological features of RDD include emperipolesis, dilated lymphatic sinuses, and foamy histiocytes. Immunohistochemically, the lesional histiocytes in RDD are typically positive for S‐100 and CD68 and negative for CD1a [8]. In this case, histologic examination revealed dilated sinuses and diagnostic emperipolesis. The IHC profile confirmed the diagnosis, showing the characteristic S‐100+/CD68+/Langerin‐ phenotype, which helps distinguish it from Langerhans cell histiocytosis (LCH).

There is no uniform treatment for RDD that is usually adapted to the actual situation of the patient. After the diagnosis, 20%–50% of nodules can shrink spontaneously, so observation can be considered [9]. Surgery for RDD is mostly used for pathological biopsies, but resection can also cure solitary diseases [1]. Radiotherapy is often used for refractory soft tissue lesions and orbital bone disease with visual impairment [10]. Therapeutic regimens for RDD remain heterogeneous. Documented successes include vincristine [11], prednisone [12], and low‐dose thalidomide [13] as single agents, with methotrexate also being effective in post‐thalidomide failure cases [14]. Use of combination chemotherapy with vincristine and prednisone has also been successful in reported cases [15]. In our case, PET‐CT showed only enlarged cervical lymph nodes with a high glucose uptake. Because the lesions were obviously enlarged and tender, we decided to administer chemotherapy instead of observation. Based on the established chemotherapeutic backbone of vincristine and prednisone for LCH [16] and considering that RDD shares certain pathological features with LCH but exhibits lower clinical aggressiveness, together with previous reports of successful prednisone monotherapy and the presentation of a solitary cervical lesion on PET‐CT in this pediatric case, we initiated first‐line treatment with oral prednisone. However, with a reduction in oral prednisone, there were no visible changes in the masses. Vincristine was then administered in combination with a gradual reduction in glucocorticoid levels. The mass significantly reduced after six doses of vincristine. We will continue to follow up on the disease status of the child.

## 4. Conclusion

RDD is rare, and the clinical manifestations of classic RDD often lack specificity. Therefore, it is recommended to obtain sufficient biopsy specimens of the lymph nodes for pathological examination to confirm the diagnosis. Treatment with prednisone and vincristine was found to be effective.

## Ethics Statement

This study was conducted in accordance with the principles of the Declaration of Helsinki. This study was approved by Tongji Medical College, Huazhong University of Science and Technology.

## Consent

Written informed consent was obtained from the patient for publication of this case report and accompanying images.

## Conflicts of Interest

The authors declare no conflicts of interest.

## Author Contributions

Jianxin Dun: conceptualization and writing the manuscript. Qun Hu: investigation and methodology. Aiguo Liu: data curation. Yaqin Wang: data curation. Ai Zhang: writing–review and editing.

## Funding

No funding was used in this study.

## Data Availability

The datasets used and/or analyzed during the current study are available from the corresponding author upon reasonable request.
